# Modulation of Dendritic Cell Apoptosis and CD8^+^ Cytotoxicity by Histamine: Role of Protein Kinase C

**DOI:** 10.1155/2017/9402814

**Published:** 2017-08-29

**Authors:** Julieta Alcain, Enrique Podaza, María Soledad Gori, Gabriela Salamone, Mónica Vermeulen

**Affiliations:** ^1^Laboratorio de Células Presentadoras de Antígeno e Inflamación, Instituto de Medicina Experimental (IMEX-CONICET), Academia Nacional de Medicina, Buenos Aires, Argentina; ^2^Laboratorio de Inmunología Oncológica, Instituto de Medicina Experimental (IMEX-CONICET), Academia Nacional de Medicina, Buenos Aires, Argentina

## Abstract

Dendritic cells (DC) are able to present extracellular antigens associated with the molecules of the major histocompatibility complex class I. In a previous work, we demonstrated that the histamine (HIS), acting through H1/H4 receptors, increases the cross-presentation of soluble ovalbumin by murine DC and can enhance the recruitment of specific CD8^+^ T lymphocytes during the development of chronic inflammatory responses. Here, we studied in more depth the mechanisms underlying this enhancement. We showed that the cytotoxicity of specific CD8^+^ lymphocytes is increased in HIS-treated DC and it is lost by inhibition of vacuolar-ATPase that prevents endosome acidification. It is known that HIS acts through G protein-coupled receptors. The H1/H4 receptors are associated with a G_q_ subunit, which involves PKC signaling, a pathway related to the apoptotic process. Interestingly, we demonstrated for the first time that HIS prevents DC apoptosis induced by heat shock through the inhibition of caspase-3, a mechanism dependent on PKC activation, since it is reversed by its inhibition. By contrast, cytolytic activity of T lymphocytes induced by HIS-stimulated DC was independent of PKC pathway.

## 1. Introduction

Dendritic cells (DC) internalize exogenous antigens (Ags) by fluid-phase pinocytosis or by receptor-mediated endocytosis [1, 2]. Peptides derived from these antigens are efficiently presented to T lymphocytes in the context of major histocompatibility complex (MHC) class II molecules. Conversely, the peptides originated in the cytosol are presented in the MHC class I. However, exogenous Ags can be presented through this pathway. Cross-presentation of antigens enables DC to present antigens associated with class I molecules, allowing the activation of CD8^+^ T responses by extracellular proteins. Antigen cross-presentation is not only relevant in the induction of antiviral and antitumor responses, but also in the induction of chronic inflammatory diseases [3]. This mechanism can be induced through two main pathways: cytosolic and vacuolar. In the first case, protein escape from the endosomal compartment allows its processing via proteasome and subsequent association with class I molecules. On the contrary, through the vacuolar pathway, the extracellular antigens are fused with the plasmatic membrane forming intracellular vesicles containing the machinery necessary to accommodate the peptides generated in class I molecules [4].

Histamine (HIS) is a main inflammatory mediator secreted primarily by mast cells. It is involved in several functions affecting neurotransmission, gastric secretion, and immunomodulation, playing a central role in the development of inflammatory pathologies such as asthma [5]. Its functions are mediated through its interaction with four histaminergic receptors (HR): H1R–H4R, which are members of the G protein-coupled receptor (GPCR) family [6]. It has been widely documented that the modulation of allergy by histamine mainly involves the H1 receptor. The use of receptor 1 antagonists proved to be successful in controlling clinical symptoms associated with pruritus and vasoconstriction in diseases such as rhinitis and conjunctivitis, but it showed no effectiveness in asthma [7, 8]. This may be explained with the fact that asthma is a multifactorial pathology; in this sense, the development of a Th2 response is essential during allergen sensitization and early inflammatory response, whereas the chronic process is strongly dependent on the recruitment of CD8^+^ Tc2 lymphocytes [9]. Gantner et al. [10] showed in humans that the interaction of histamine with H2R and H4R induces the secretion of IL-16 by CD8^+^ T lymphocytes, which determines the migration of CD4^+^ and CD8^+^ T cells to lung tissues. Also, in a model of contact dermatitis, the induction in the skin of IL-17^+^ CD8^+^ T lymphocytes was shown to be mediated by HIS via the H4R [11].

Moreover, contradictory results were reported in relation to the pro- or antiapoptotic effect of HIS. Thus, in human monocytic cells, Soga et al. [12] demonstrated that it prevents apoptosis via the upregulation of Bcl-2 and Mcl-1 and the suppression of activated caspase-3, an effect dependent on H2R signaling. On the other hand, in the human embryonic cell line H3K293 transfected with the enzyme histidine decarboxylase (HDC), responsible for HIS synthesis [13], its production was associated with the upregulation of the proapoptotic effector caspase-3 and cell cycle arrest by alteration in proteins such as cyclin D1 and A1.

Here, we study the intracellular mechanisms triggered by the HIS which determine DC functionality. We found that HIS prevents the apoptosis of DC, as shown by the inhibition of procaspase-3 and caspase-3. Interestingly, this mechanism depends on PKC activation. We also show that DC cross-presentation of antigen induced by HIS involves the vacuolar pathway resulting in the induction of a specific cytotoxic response. However, this activity is not dependent on PKC activation.

## 2. Materials and Methods

### 2.1. Mice

All experiments were carried out using 2-month-old virgin female C57BL6/j mice and OT-I mice on the C57BL6/j background raised at the National Academy of Medicine, Buenos Aires, Argentina. They were housed six per cage and kept at 20 ± 2°C under automatic 12 h light-dark schedule. Animal care was in accordance with institutional guidelines.

### 2.2. DC Generation from Bone Marrow Cultures

The procedure used to obtain DC was described by Inaba et al. [14], with minor modifications [15]. Briefly, bone marrow was flushed from the limbs of long bones using 2 ml of RPMI 1640 (Invitrogen, Carlsbad, CA) with a syringe and 25-gauge needle. Red cells were lysed with ammonium chloride (0.45 M). After washing, cells were suspended at a concentration of 1.5 × 10^6^ cells/ml in 80% RPMI 1640 medium supplemented with 10% fetal calf serum, and 5.5 × 10^−5^* β*-mercaptoethanol (Sigma) (complete medium) and 20% J588-GM cell line supernatant. The cultures were fed every 2 days by gently swirling the plates, aspirating 50% of the medium, and adding back fresh medium with J588-GM cell line supernatant. On day 9 of culture, more than 90% of the harvested cells expressed MHC class II, CD40, and CD11c, but not Gr-1 (not shown).

### 2.3. Flow Cytometry

The following monoclonal antibodies (mAbs) were used, conjugated with fluorescein isothiocyanate (FITC), phycoerythrin (PE), or peridinin chlorophyll protein complex (PerCP): anti-CD11c, anti-H2K^b^, anti-GR1, anti-CD8, the lysosomal membrane proteins CD107a (LAMP-1) (BD Pharmingen), and the OVA 257-264 (SIINFEKL) peptide bound to H2K^b^ antibody (25D1.16; eBiosciences). To perform staining, cells were incubated with the corresponding conjugated antibody, or isotype-matched control antibody, for 20 min. Finally, cells were washed once again and analyzed by flow cytometry. Data were collected using a FACScalibur flow cytometer and analyzed with CellQuest software (Becton Dickinson, San Jose, CA).

### 2.4. Confocal Microscopy

DC were treated with or without HIS for 20 min and then incubated in the presence of OVA-FITC (Sigma) for 1 h at 37°C. After washing, cells were attached in L-polylisine pretreated coverslips and blocked with decomplemented normal mice serum 0.1% in RPMI medium for 1 hour at 37°C. Cells were washed twice with PBS and then fixed with 2% of paraformaldehyde (Merck, Germany) for 20 minutes at 4°C. The fixed cells were washed twice with PBS. DC were incubated with PE-conjugated antibodies directed to MHC class I (H2K^b^; BD Pharmingen) for 20 min at room temperature. Finally, after washing, coverslips were mounted onto microscope slides using Fluoromount medium (Vector Labs; Southfield; MI, USA). Images were taken using a FluoView FV1000 confocal microscope (Olympus, Tokyo, Japan). Analysis was performed with Olympus FV10-ASW software.

### 2.5. Western Blot Analysis

DC were suspended in RPMI 10% fetal calf serum (4 × 10^6^/ml) and treated with or without HIS (0.1 *μ*M) for 10 min at 37°C, then washed with PBS. Pellets were resuspended in Western blot loading buffer (100 mM Tris-HCl pH 6.8; 4% SDS, 0.2% bromophenol blue, 20% glycerol, and 200 mM dithiothreitol), heated for 5 min at 95°C and frozen at −80°C. Proteins were separated onto 10% SDS-PAGE followed by electroblotting. The membranes were blocked in PBS + 5% milk powder for 1 h and then incubated with the following primary antibodies in blocking buffer + 0.05% Tween 20 overnight at 4°C: anti-phospho-P38K (1 : 1000; Santa Cruz Biotechnology), anti-phospho-Akt (1 : 300; Santa Cruz Biotechnology), anti-caspase-3 (1 : 2000; Cell Signaling Technology), anti-caspase-9 (1 : 500, Cell Signaling Technology), and anti-phospho-(Ser) PKC (1 : 100; Cell Signaling Technology). After washing, secondary antibodies were applied in blocking buffer for 1 h at room temperature: anti-rabbit, anti-mouse, or anti-goat mAb-HRP (1 : 2000; Cell Signaling Technology). Specific bands were developed by enhanced chemiluminescence (ECL, Amersham Biosciences, Uppsala, Sweden). Membranes were stripped and reprobed with a rabbit mAb against murine *β*-actin (1 : 3000; Cell Signaling Technology). The quantification was performed using the ImageQuant program.

### 2.6. Cytotoxicity Assay

#### 2.6.1. In Vivo

Cytotoxicity was performed as described previously. In brief, bone marrow-derived DC were loaded with OVA (100 *μ*g/ml) and treated with or without HIS. HIS-DC were labeled with 5 nM (CFSE^high^) fluorescent dye carboxyfluorescein succinimidyl ester (CFSE, Invitrogen). Ct-DC were labeled with 0.5 nM CFSE (CFSE^low^). Then, CFSE^high^ and CFSE^low^ cells were mixed in a 1 : 1 ratio (1 × 10^7^ cells/population) and inoculated intravenously (i.v.) into naïve syngeneic mice which were previously immunized i.v. with OVA (100 *μ*g/ml). The number of CFSE^+^ cells remaining in the spleen after 4 hours was determined by flow cytometry. Cytotoxicity (Cx) was expressed as the percentage of lysis, calculated as [1 − (*r*_immune_/*r*_control_)] × 100, where *r* is given by the expression of %CFSE^high^/%CFSE^low^ cells for immune and nonimmunized (control) mice, respectively. In some experiments, we used OT-I mice to evaluate the Cx.

#### 2.6.2. In Vitro

OVA-loaded (100 *μ*g/ml) DC were stained with 5 *μ*M of CFSE for 40 min at 37°C, and after exhaustive washing, cells were incubated in the presence of splenic mononuclear cells from OVA-immunized mice (ratio 1 : 4, DC and mononuclear cells, resp.) for different times. After this, the percentage of CFSE^+^ cells in the cocultures was determined by flow cytometry and compared to the percentage of CFSE^+^ cells obtained from nonimmunized mice.

### 2.7. Apoptosis Assay

To evaluate apoptosis, we used the Annexin-V-propidium iodide (PI) approach. The assay consists in the fluorescent labeling of phosphatidylserine groups expressed on the extracellular side of apoptotic cell membrane. Also, to assess nuclear integrity, we used propidium iodide. For this, DC (5 × 10^5^), stimulated or not with HIS (0.1 *μ*M) and inhibited or not with bafilomycin (Baf; 0.1 *μ*M) and Gö6983 (protein kinase C inhibitor (PKCi), 2 nM), were exposed to heat shock (20 minutes, 42°C) to induce apoptosis. Then, at different time points, cells were stained with Annexin-V-FITC (1 *μ*g/ml in binding buffer) for 20 minutes at room temperature. At the end of the incubation time, we added PI (3 *μ*g/ml) and the samples were immediately analyzed by cytometry. Both Annexin-V^+^ and double-positive Annexin-V^+^ PI^+^ cells were considered apoptotic.

### 2.8. Determination of Cytokines

Cytokine levels in supernantants of cultured DC exposed to 42°C for 24 h were measured by ELISA. IL-12, TNF-*α*, and MCP-1 (eBiosciences, San Diego, CA) were performed according to the manufacturer's protocols. The detection limits were 4 pg/ml for IL-12, 8 pg/ml for TNF-*α*, and 12.5 pg/ml for MCP-1.

### 2.9. Statistical Analysis

Differences among treatments were determined by one-way ANOVA, followed by post-ANOVA comparisons using the Tukey's test. Differences between two means were analyzed using Student's *t*-test. A *p* value < 0.05 was considered to indicate statistical significance.

## 3. Results

### 3.1. Histamine Increases Class I Presentation via Modulation of Vacuolar Pathway

**Figure 1:**
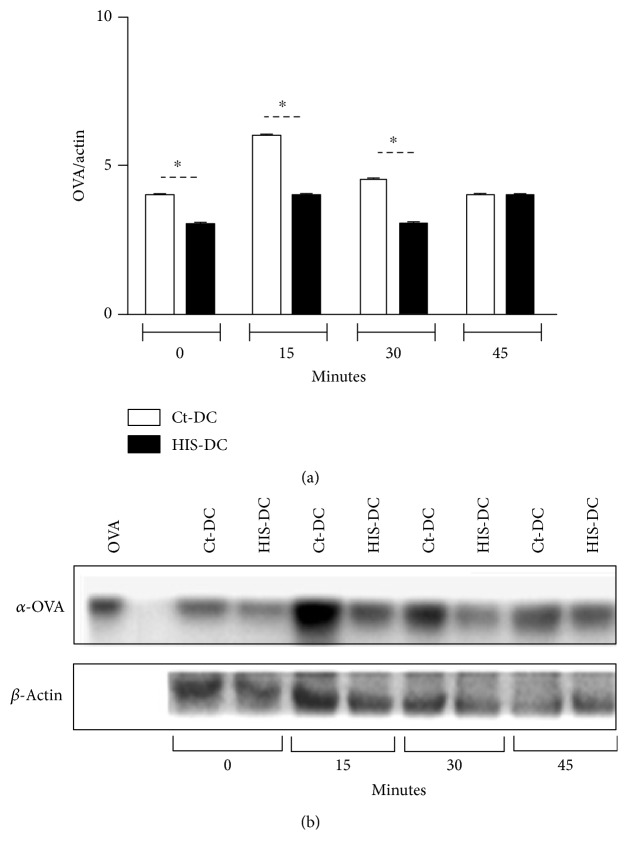
Histamine increases OVA-degradation timing. In (a), we show the ratio of OVA/*β*-actin at different times after HIS (0.1 *μ*M, 37°C) stimulation. Bars represent the mean ± SE, *N* = 4 experiments (^∗^*p* < 0.05). In (b), a representative Western blot is shown.

Previously, we demonstrated that histamine is able to increase the presentation of soluble antigens via MHC class I [16]. Here, we analyze in more depth the mechanisms involved. In the first place, DC were treated with HIS (HIS-DC; 0.1 *μ*M, 30 min at 37°C) or without HIS (Ct-DC) and finally incubated with OVA (100 *μ*g/ml). At different times, lysates were obtained and the generated peptides were evaluated by Western blot. As shown in Figures 1(a) and 1(b), the kinetics of antigen processing or degradation indicates that this process is faster for DC stimulated with HIS between 15 and 30 min, but after the 30 min, no significant differences in degradation rate can be observed between treatments. In fact, Ackerman and Cresswell [17] associated a rapid degradation to class I presentation.

**Figure 2:**
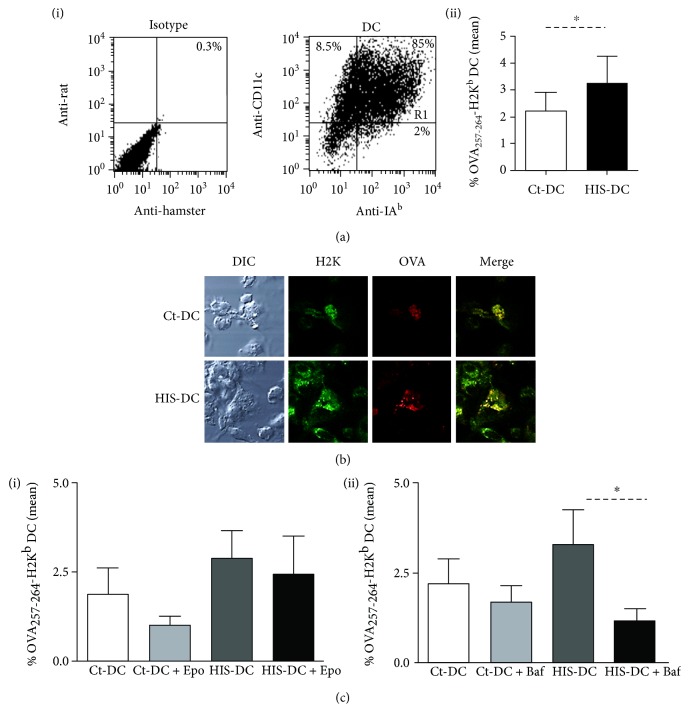
Histamine modulates presentation via the vacuolar pathway. (a), (i) A representative dot plot of DC region used for the analysis is shown. (a), (ii) The mean percentage of OVA257-264-H2K^b+^ DC is significantly higher for HIS-stimulated DC. (b) We show the colocalization at the membrane of DC of class I molecules and the generated OVA epitope. (c) DC were incubated with Epo or Baf for 90 min at 37°C and then treated or not with HIS (0.1 *μ*M). After 20 min, cells were incubated in the presence of OVA-FITC (100 *μ*g/ml). Finally, after 2 h of culture, DC were washed and stained with anti-mouse SIINFEKL-H2K^b^ for 20 min at 4°C. Bars represent the % mean of OVA_257–264_-H2K^b+^ cells ± SE of 8 independent experiments for DC inhibited with Epo (i) and Baf (ii) (^∗^*p* < 0.05, ANOVA and Tukey's test).

Next, we decided to evaluate the possible pathways modulated by HIS on dendritic cells. In a first set of experiments, DC treated with or without HIS were incubated with OVA-FITC (100 *μ*g/ml). Then, cells were stained with the antibody anti-OVA (peptide 257-264; SIINFEKL) joined to the class I molecule (H2K^d^). The percentage of DC (region R1 of positive CD11c-IA^b^ cells, Figure 2()(a), (i)) that stained positive to OVA_257-264_ peptide bound to H2K^d^ antibody at membrane level was analyzed by flow cytometry. As shown in Figure 2(a), (ii), the percentage of double-positive cells was increased in a 50% for HIS-DC than Ct-DC. To corroborate this result, we analyzed OVA presentation by confocal microscopy. In Figure 2(b), we observed the colocalization of H2K-OVA_257–264_ complexes in the membrane of DC, being greater for HIS-stimulated cells than for nonstimulated DC. Finally, before their stimulation with HIS, we preincubated cells with the inhibitors of the main pathways involved in class I presentation, bafilomycin (Baf) (vacuolar pathway) and epoxomicin (cytoplasmatic pathway). As shown in Figure 2(c), (i), the incubation of DC with epoxomicin (1 ng/ml), a proteasome inhibitor, did not modify the percentages of antigenic presentation in DC treated with HIS. It should be noted that, as expected, double-positive staining was slightly inhibited in untreated controls. Then, we analyzed this phenomenon in the presence of Baf (0.1 *μ*M), an inhibitor of vacuolar proton pump (v-ATPase), needed for the reduction of vacuolar pH resulting in the antigenic degradation [18]. We showed in Figure 2(c), (ii) that Baf significantly reduced cross-presentation of HIS-DC by 65% compared to DC. In a preliminary assay, we also observed that inhibition of cathepsin S (CatSi) activity decreases almost by 74% of the class I presentation by HIS-DC stimulated (Ct-DC: 3.9 ± 0.42; CatSi-DC: 2.1 ± 0.19; HIS-DC: 6.1 ± 0.55; HIS-DC + CatSi: 1.6 ± 2.2; *N* = 2).

### 3.2. Histamine Enhances Cytotoxicity Activity of Specific T-CD8 Lymphocytes

**Figure 3:**
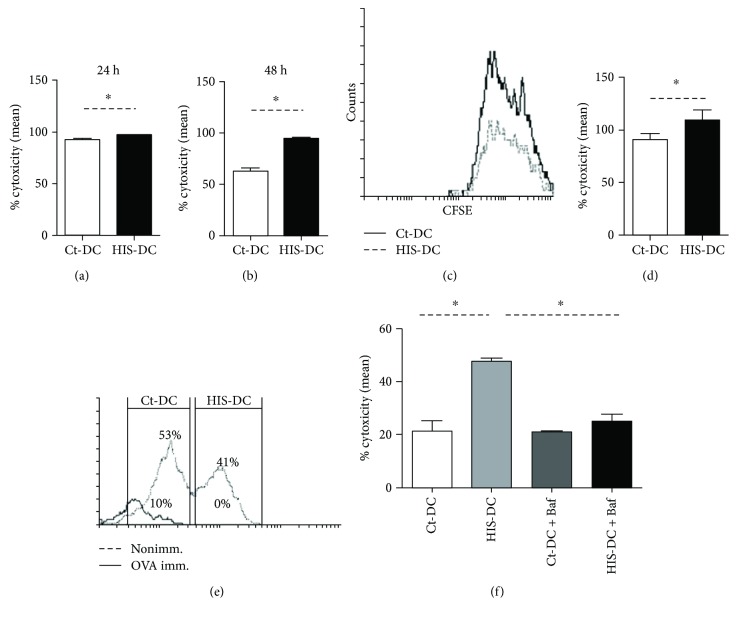
Histamine increases the ability of DC to activate CD8^+^ cytotoxicity. Histograms show the cytotoxicity after 24 h (a) and 48 h (b) of *in vitro* coculture of OVA-immunized splenic mononuclear cells (5 × 10^6^) with OVA loaded-DC stimulated or not with HIS (1.25 × 10^5^). Bars represent the mean Cx percentage ± SE of 6 independent experiments. In (c), we show a representative *in vitro* Cx histogram plot. The cytotoxicity activity *in vivo* was evaluated in splenocytes obtained after 4 h of OVA loaded DC transfer (1 × 10^7^ cells, injected i.v. to OVA-immunized mice). In (d), the bars represent the mean percentage ± SE of 5 independent *in vivo* experiments. *p* < 0.05, Student's *t*-test. (e) A representative histogram is shown. (f) 10^7^ loaded DC were transferred to OTI-mice after its treatment with Baf (0.1 *μ*M) for 90 min at 37°C. Then, DC were treated or not with HIS (0.1 *μ*M). Bars represent the mean cytotoxicity percentage ± SE of 5 independent experiments (^∗^*p* < 0.05, ANOVA Tukey's test).

The above results show that HIS promotes extracellular antigen presentation in the context of MHC class I. Next, we decided to test whether this HIS modulation affects the CD8^+^ T cells activity and, consequently, increases cytolytic activity. To this aim, cytotoxicity (Cx) was evaluated. The mononuclear spleen cells from immunized mice were cocultured with OVA-loaded DC stained with CFSE (5 nM) and finally incubated for 24 or 48 h as described in Materials and Methods. As shown in Figures 3(a), 3(b), and 3(c), a significantly higher lytic activity was observed when DC were stimulated with HIS compared to Ct-DC at the two time points analyzed, although it is more significant at 48 h (approximately 50% increase). Strikingly, in the *in vivo* assays, after 4 h of DC transfer, the Cx activity was significantly greater (19% increment) when cells were preincubated with HIS (Figures 3(d) and 3(e)). Finally, since Baf inhibited DC class I presentation induced by HIS, we decided to evaluate the cytotoxicity in the presence of this vacuolar ATPase inhibitor. With this aim, DC were transferred to OTI mice after their loading with the H2K^b^-binding peptide OVA 257-264 (SIINFEKL, 1 *μ*mol/l). As shown in Figure 3(f), the inhibition of vacuolar pathway using Baf reduced significantly by 47.8% the Cx induced by HIS acting on DC.

**Figure 4:**
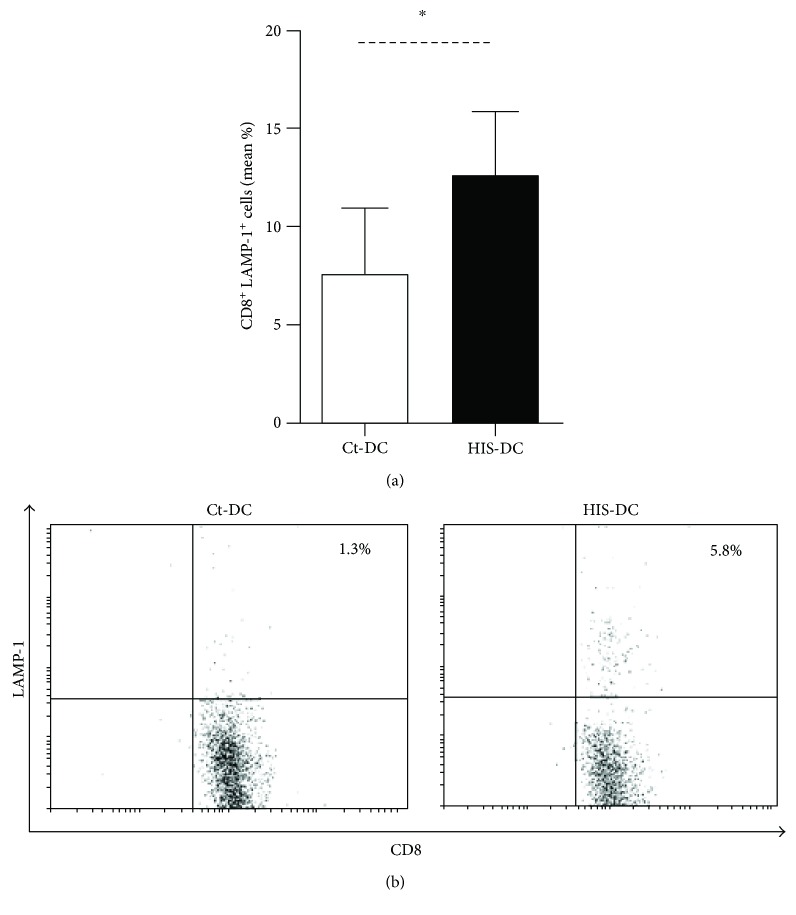
CD8^+^ T lymphocyte degranulation is higher in cocultures with HIS-treated DC. Degranulation was assessed by LAMP-1 fluorescent staining and flow cytometry. (a) Bars represent the mean LAMP^+^CD8^+^ percentage ± SE of 4 experiments. ^∗^*p* < 0.05, Student's *t*-test. (b) A representative dot plot is shown.

In addition, we performed the staining of LAMP-1, a marker of CD8^+^ T lymphocyte degranulation [19]. In Figures 4(a) and 4(b), the percentage of double-positive CD8/CD107a obtained by flow cytometry is shown. This shows that a higher number of CD8^+^ T cells were degranulated in the presence of HIS-stimulated DC; thus, HIS can modulate the ability of DC to induce CD8 cytotoxicity.

### 3.3. Histamine Interaction with Dendritic Cells Activates PKC and Caspase Pathway

**Figure 5:**
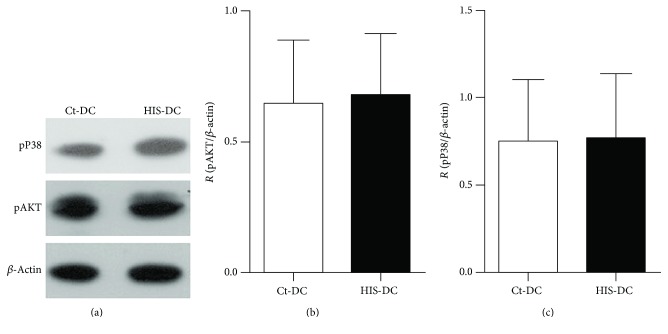
Histamine does not have an effect in Akt or P38 phosphorylation. DC were stimulated or not with HIS (0.1 *μ*M) and protein expression was analyzed by Western blot in lysates obtained after 10 min of incubation at 37°C. In (a), we show a representative gel. The histograms represent the quantification of Akt (b) and pP38 (c) expressed as the protein/*β*-actin ratio. Bars represent the means ± SE, *n* = 4.

To study which pathways are activated by HIS action on DC, we used the Western blot technique after DC stimulation with HIS for 10 minutes. First, we studied Akt and P38 proteins, since they are known to be involved in DC functionality [20–22]. As shown in Figures 5(a), 5(b), and 5(c), these pathways are not modulated by HIS.

**Figure 6:**
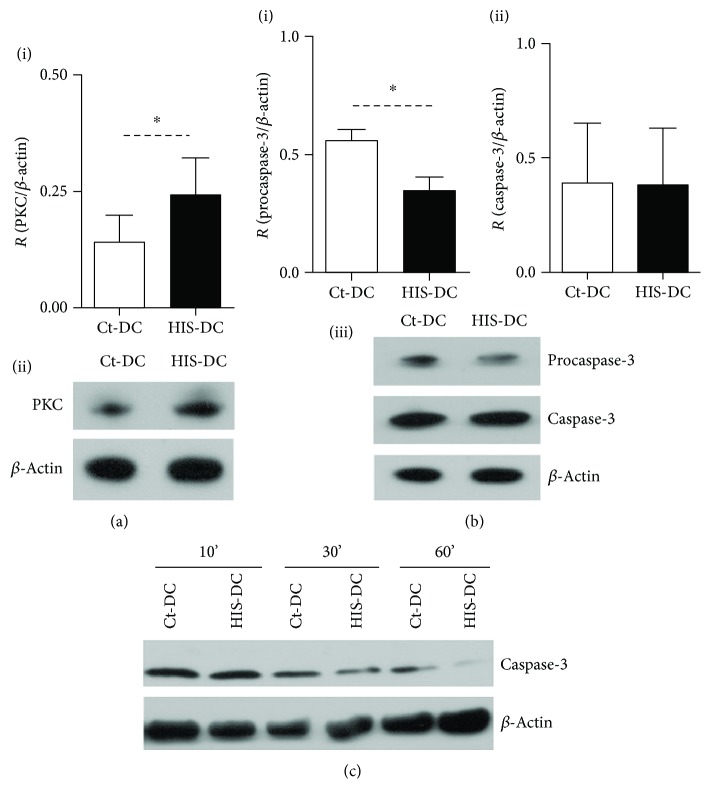
Histamine modulates PKC and caspase activation. (a), (i) Bars represent the mean ± SE of 8 experiments (^∗^*p* < 0.05, Student's *t*-test). (a), (ii) A representative Western blot of PKC is shown. In (b), (i) and (ii), we show the quantification of procaspase-3 and caspase-3, respectively. Bars represent the mean ratio ± SE of 5 experiments, ^∗^*p* < 0.05, Student's *t*-test. (b), (iii) We show a representative gel for procaspase-3 and cleaved caspase-3. (c) Analysis of cleaved caspase-3 at different time points was performed. We show one representative blot.

Then, as PKC is also associated to GPCR, such as HIS receptors, we evaluated PKC activation in HIS-treated DC.  Figure 6(a), (i-ii) shows that PKC is significantly activated after 10 min of stimulation. Since PKC phosphorylation is associated to apoptosis and caspase-dependent cytotoxicity mechanisms [23, 24], we analyzed the expression of caspase-3 and caspase-9 on DC. Interestingly, in HIS-treated DC, the expression of procaspase-3 was significantly reduced, although this decrease was not related to a modification of effector caspase-3 levels (Figure 6(b), (i-ii)), while the analysis of caspase-9 showed no difference between treatments (data not shown). When the cleaved caspase-3 was evaluated at different time points, we observed a significant reduction of this protein in HIS-stimulated cells compared to Ct-DC at longer stimulation times (Figure 6(c)).

### 3.4. PKC Activation Is Implicated in Cellular Death

**Figure 7:**
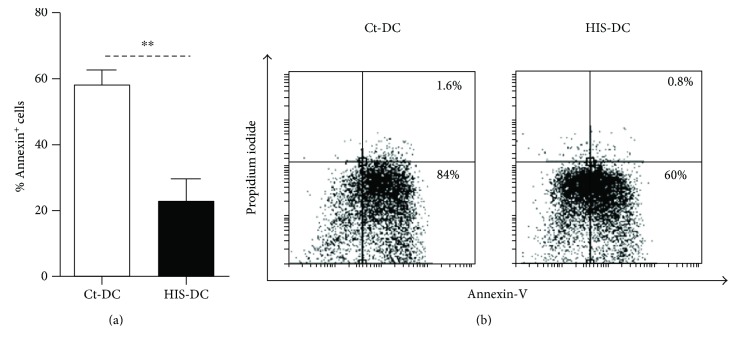
Histamine prevents DC apoptosis. We performed an assay with Annexin-V and propidium iodide. (a) Bars represent mean percentage Annexin^+^ cells ± SE, *n* = 5. ^∗∗^*p* < 0.05, Student's *t*-test. (b) A representative dot plot is shown.

Taking into account our previous results and the fact that HIS induction of neutrophil apoptosis is dependent on PKC phosphorylation via the caspase-3 pathway [25], we decided to study the possible effects of HIS on the modulation of DC apoptosis. Apoptosis induced by heat shock at 42°C was evaluated in Ct-DC and HIS-DC. After 24 h, the apoptosis was assessed by cytometry using Annexin-V-propidium iodide staining. As shown in Figures 7(a) and 7(b), the pretreatment of cells with HIS prevents their apoptosis by 30%.

**Figure 8:**
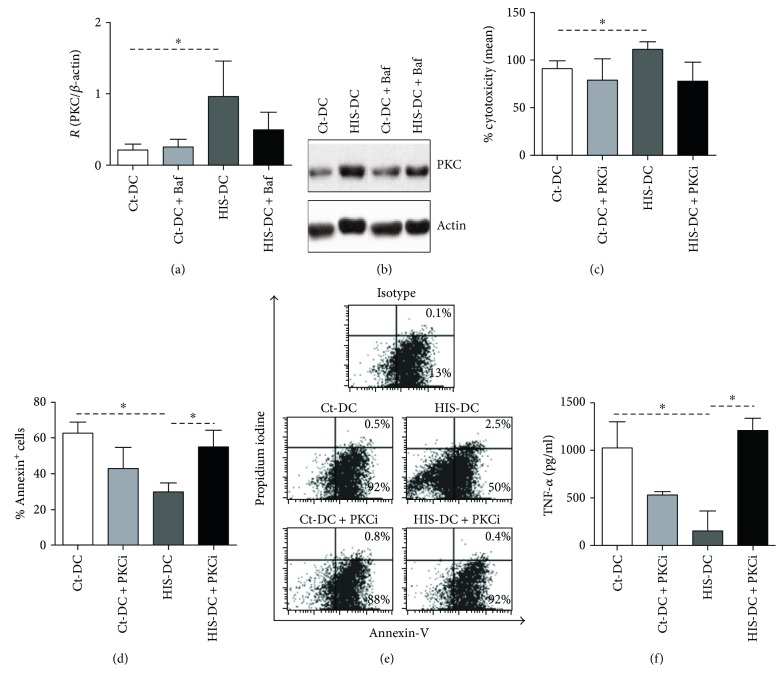
PKC modulates the apoptosis of DC stimulated by HIS. DC were treated with or without Baf (0.1 *μ*M). After 90 min, DC were stimulated or not with HIS (0.1 *μ*M). In (a), bars represent the quantification of PKC as the mean ± SE of 5 obtained from Western blot experiments, *p* < 0.05, ANOVA and Tukey's test. In (b), a representative gel is shown. (c) Histograms show the cytotoxicity after 48 h of *in vitro* coculture of OVA-immunized splenic mononuclear cells (5 × 10^5^) with OVA loaded-DC stimulated or not with HIS (1.25 × 10^5^). Bars represent the mean of cytotoxicity percentage ± SE of 6 independent experiments. *p* < 0.05, ANOVA, and Tukey's test. In figure (d), after inhibition of PKC pathway, HIS-stimulated DC were exposed to 42°C. Finally, apoptosis was analyzed 24 h later by flow cytometry. In (d), bars represent the mean ± SE, *n* = 5 (*p* < 0.05, ANOVA Tukey's test). In (e), a representative dot plot is shown with Annexin-V and propidium iodide. (f) TNF-*α* production in DC cultures was analyzed in the supernatants of apoptotic cells at 24 h by ELISA. The bars represent the mean ± SEM of 4 experiments. The figure shows mean concentration values (pg/ml). Asterisks indicate statistical significance (^∗^*p* < 0.05, ANOVA and Tukey's posttest).

Because HIS activates PKC pathway, we decided to evaluate its functional role. First, we analyzed PKC expression in DC treated with HIS in the presence of Baf. As demonstrated in Figures 8(a) and 8(b), the blockage of vacuolar pathway does not affect PKC activation and therefore does not modulate cytotoxic activity (Figure 8(c)). On the other hand, apoptosis was inhibited by 85% when PKC phosphorylation was blocked (Figures 8(d) and 8(e)). Finally, we evaluated the secretion of inflammatory cytokines in supernatants of apoptotic cells.  Figure 8(f) shows that PKC activity blocking in HIS-DC significantly increases TNF-*α* production while MCP-1 and IL-12 were not affected (data not shown).

## 4. Discussion

Although in many previous works it has been proved that histamine is able to induce the recruitment or activation of CD8^+^ T cells to skin and lung among others [10, 26], little is known regarding the mechanisms involved. In our laboratory, we demonstrated previously that HIS increases class I presentation by murine DC [27]. Here, we evaluate the mechanisms responsible for this effect and, surprisingly, we found that the effect was independent of proteasome activity. On the contrary, in DC stimulated with HIS, the antigen was retained in the vacuolar compartment. As it was observed in the presence of bafilomycin, which prevents vacuolar acidification, the generation of antigenic peptides for their subsequent accommodation in the class I molecules was compromised. In fact, Rock and Shen [28], many years ago, showed that in the vacuolar compartment exists the complete machinery and specific proteases which allow the generation of peptides for class I molecules. One of the proteases involved is cathepsin S; however, in our system, we cannot rule out the participation of other proteases, since the inhibition only reduced by 74% the expression of the MHC I-OVA complexes on the cellular surface. It is clear that other proteases and mechanisms could be also involved in the enhancement of dendritic cell presentation by histamine.

We prove for the first time that HIS prevents apoptosis of murine DC. Recently, Deng et al. [29] described in humans and mice that HIS increases during myocardial infarction. Interestingly, the amine depletion contributes to heart disease through the inhibition of macrophage recruitment and increase of cardiomyocyte apoptosis. Furthermore, via its interaction with H2R, HIS prevented the apoptosis of NK cells, mediated by inactivation of NADPH oxidase [30]. In our system, HIS was able to inhibit the levels of both the inactive and active forms of caspase-3 in DC. In a model of carrageenan-induced pleurisy in rats, the induction of an inflammatory response was associated with the recruitment of leukocytes and nitric oxide production, which together with the activation of caspase-3 upregulated oxidative damage and apoptosis [31]. The use of an H4R antagonist reversed dramatically the tissue injury. However, an interesting fact was described by Matsuda et al. [32] in a model of sepsis, where the upregulation of H4R was responsible for spleen T lymphocyte apoptosis. We found this data encouraging because our model showed a delay in DC programmed death but an increase of cytotoxicity induced by specific T lymphocytes, and both mechanisms could be mediated by H4R. In fact, preliminary results of our group demonstrated that H4R inhibition using thioperamide results in downregulation of cytolytic activity induced by HIS (data not shown).

The interaction of HIS with DC triggers the phosphorylation of PKC protein. PKC comprises several serine/threonine kinases determining three isoform families (conventional, novel, and atypical) which differentially depend on second messengers associated for their activation [33]. This protein kinase has a main role in neurotransmission, gene regulation, growth, differentiation, and apoptosis. It was shown that induction of apoptosis by histamine involves the activation of conventional isoforms (*α*, *β*, and *γ*) [34]) that require Ca^++^ activation, a second messenger associated to the histamine receptors signaling (7). Also, high concentrations of histamine, such as those associated to inflammatory foci, mediate the apoptosis of neutrophils through caspase and the novel PKC *δ* activation [25]. Since our antibody reveals both conventional and novel isoforms, experiments are in progress with specific antibodies to determine the PKC family involved in DC apoptosis induced by histamine.

The upregulation of PKC was described during H1R signaling, generally associated with the modulation of the production or release of neurotransmitters such as catecholamine and acetylcholine [35, 36]. Moreover, this intracellular pathway has a central role in the modulation of epithelial barrier, nervous control of arterial contraction/dilatation [37, 38], and lung tissues. Thus, bronchial epithelial cells are able to secrete inflammatory cytokines and growth factors, through the stimulation of H1R by HIS [39]. In this regard, we showed that HIS-stimulated DC inhibits the secretion of proinflammatory cytokines such as TNF-*α*, a known inducer of cellular apoptosis [30], after heat shock. On the other hand, it has been proved that HIS on keratinocytes and on epithelial cells activates PKC signaling and induces the secretion of the antiapoptotic factor GM-CSF [40, 41].

## 5. Conclusion

Summarizing, our results show for the first time that HIS has the ability to prevent the normal apoptosis program during the perception of damage signals by DC and that the interaction of HIS with DC favors the increase of soluble antigens presentation in the context of class I molecules mediated by the vacuolar pathway activation, culminating with the improvement of specific lytic response by specific CD8^+^ T lymphocytes.

A future approach will determine whether blocking vacuolar pathway or PKC activation could improve the current therapies used in inflammatory pathologies such as asthma or dermatitis, where the induction/recruitment of CD8^+^ T lymphocytes correlates with chronic phase of disease.

## Abbreviations

DC: Dendritic cells
PKC: Protein kinase C
Cx: Cytotoxicity
HIS: Histamine
Ags: Antigens
HRs: Histamine receptors
MHC: Major histocompatibility complex.
